# Self–other recognition impairments in individuals with schizophrenia: a new experimental paradigm using a double mirror

**DOI:** 10.1038/s41537-018-0065-5

**Published:** 2018-11-28

**Authors:** Gaelle Keromnes, Tom Motillon, Nathalie Coulon, Alain Berthoz, Foucaud Du Boisgueheneuc, Moritz Wehrmann, Brice Martin, Bérangère Thirioux, Olivier Bonnot, Romain Ridereau, Eric Bellissant, Dominique Drapier, David Levoyer, Nemat Jaafari, Sylvie Tordjman

**Affiliations:** 1 Pôle Hospitalo-Universitaire de Psychiatrie de l’Enfant et de l’Adolescent, Université de Rennes 1 and Centre Hospitalier Guillaume Régnier, 154 rue de Châtillon, Rennes, France; 2 Laboratoire Psychologie de la Perception, Université Paris Descartes and CNRS UMR 8242, Paris, France; 3 CHU de Brest – Department of Psychiatry, UBO and Hôpital de Bohars, Bohars, France; 4 Laboratoire de Physiologie de la Perception et de l’Action UMR 7152 CNRS, Collège de France, Paris, France; 5 Département de Neurologie, Centre de Mémoire de Ressource et de Recherche, CHU de Poitiers, Poitiers, France; 6 Bauhaus-Universität Weimar, Weimar, Germany; 7 Centre Référent Lyonnais en Réhabilitation et en Remédiation Cognitive - Service Universitaire de Réhabilitation, Hôpital du Vinatier, Université Lyon 1, et UMR, 5229 Lyon, France; 8 Unité de Recherche Clinique Intersectorielle en Psychiatrie à vocation régionale Pierre Deniker, Centre Hospitalier Henri Laborit, Poitiers, France; 9 Department of Child and Adolescent Psychiatry, Nantes University Hospital, Nantes, France; 10 INSERM, CIC 1414 Clinical Investigation Center, Rennes, France; 11 Pôle Hospitalo-Universitaire de Psychiatrie de l’Adulte, Université de Rennes 1 and Centre Hospitalier Guillaume Régnier, Rennes, France; 12 Université de Poitiers – INSERM CIC 1402, CHU de Poitiers – INSERM U 1084, Experimental and Clinical Neuroscience Laboratory – Groupement de Recherche CNRS 3557, Poitiers, France

## Abstract

Clinical observations suggest early self-consciousness disturbances in schizophrenia. A double mirror combining the images of two individuals sitting on each side of the mirror was used to study self–other differentiation in 12 individuals with early onset schizophrenia (EOS) and 15 individuals with adult onset schizophrenia (AOS) compared to 27 typically developing controls (TDC) matched on age and sex. The effects of intermodal sensory perception (visual–tactile and visual–kinesthetic) on self–other recognition were also studied. The results showed that EOS and AOS individuals, independently of age and schizophrenia severity, were centered on their own image compared to TDC, with both significant earlier self-recognition and delayed other-recognition during the visual recognition task. In addition, there was no significant effect of intermodal sensory stimulation on self–other recognition in EOS and AOS patients, whereas self-centered functioning was significantly increased by visual–tactile stimulation and decreased by visual–kinesthetic stimulation in TDC. The findings suggest that self–other recognition impairments might be a possible endophenotypic trait of schizophrenia.

## Introduction

Early diagnosis of schizophrenia and detection of patients at high or ultrahigh risk of schizophrenia are current important issues in psychiatry. However, the conventional diagnostic criteria of schizophrenia, as proposed in the Diagnostic and Statistical Manual of Mental Disorders, Fifth Edition (DSM-5)^1^ and International Classification of Diseases (ICD-10)^2^ show very low sensibility and a lack of reliability for early diagnosis of schizophrenia.^3,4^ Among the reasons that could explain these difficulties (especially in the context of early onset schizophrenia, EOS), some authors^5^ have suggested that DSM and ICD diagnostic criteria have simplified the clinical features of schizophrenia and consequently capture only a fragment of the clinical core of schizophrenia. More precisely, alterations of subjective experience, such as distortions of the perception of self are neglected, although alterations of the sense of self in schizophrenia have been reported in most seminal texts^6,7^ as well as in phenomenological descriptions of schizophrenia.^8,9^ Schizophrenia is nowadays considered as a disorder of the self.^10^ Self-consciousness disturbances in schizophrenia are various and can include different problems such as altered body self-consciousness, agency impairments, or social cognition disorders.^11,12^ According to several authors, self-consciousness disturbances are expressed in schizophrenia through notably difficulties of *self–other differentiation*.^13,14^ Identification of such disturbances might be clinically useful for identifying individuals with schizophrenia or at risk of schizophrenia.

Schizophrenia is a chronic psychiatric disorder with a prevalence of approximatively 0.3%–0.7%^1^ and typically adult onset schizophrenia (AOS, at age ≥18 years). The most common definition of EOS (prevalence of approximatively 0.03%^15^) and very early onset schizophrenia (VEOS, prevalence of approximatively 0.002%^15^) is, respectively, schizophrenia with onset before age 18 years and schizophrenia with onset before age 13 years.^15,16^ Research on EOS and VEOS remains limited due to their low prevalence and their lack of specificity in the DSM-5 or ICD-10 diagnostic classifications.^16^ A better understanding and detection of EOS and VEOS is nonetheless necessary, considering that early diagnosis and treatment of schizophrenia are associated with better prognosis.^17^

Self-consciousness allows both self-recognition and self–other differentiation and is the basis of social interactions. Self-consciousness develops through a long process that starts in the first years of life and is reworked throughout an individual's life. Paul Schilder^18^ proposed the concept of *body image* to designate the way a person recognizes his or her body and face as being his or her own. The term *body image* may be considered restrictive because the concept of self is not only limited to visual perception. Ulric Neisser^19^ described two different perceptual aspects of self-development, first through body perceptions and interactions with external objects, and second through the relation to others, based on ideas already developed by Henri Wallon.^20,21^ Wallon has observed and studied how individuals, from an early age, interact with their environment through the body^20^ and use mirrors as a support of self-recognition.^21^ René Zazzo,^22^ inspired by Wallon, observed very young children and their reactions to their own images. He described, notably, the way in which recognition of others (acquired from 8 months) precedes by far self-recognition (acquired at approximately 2 years old) across different visual media (mirror, photo, film) with progressive awareness of the own body image concurrently with language development. This suggests that language development, in terms of its social communication dimension, requires self–other differentiation. More recently, these models and ideas were re-examined and enriched by several authors such as Damasio, Rochat, Decety, and Sommerville (Table 1).^23–25^ Other authors focused on the comprehension of the sensory component of self-consciousness and the key role of the body as the interface between self and the environment.^26,27^ They also described how body self-consciousness can vary depending on the sensory stimuli to which the body is exposed.^28^ The body becomes as much a component of self-consciousness (body self), as a receptacle of diverse sensory information that facilitates the development of self-consciousness over the lifespan.

**Table 1:** Conceptualizing the self (based on Damasio,^23^ Rochat,^24^ Decety,^25^ and Sommerville^25^)

	Consciousness	
Levels of consciousness^a^	Pre-reflexive consciousness (implicit)	Early appearance, relies on bodily perception
	• Level 1: Differentiation	• Relies on the experience of own bodily movements
	• Level 2: Situation	• Relies on intermodal sensory perception of the own body
	Reflexive consciousness (explicit)	The self is expressed explicitly
	• Level 3: Identification	• Identification of the self in the mirror
	• Level 4: Permanence	• Identification of a permanent self (invariant over time) in pictures and movies
	Self-consciousness (explicit)	Later appearance, relies on mental representations
	• Level 5: “Meta” self-awareness	• Notably, representations of how the child is perceived by others
Type of consciousness	Agency	Consciousness of volition and ownership
	Distinctiveness	Consciousness of uniqueness
	Personal continuity	Consciousness of continuity through time
	Reflection	Consciousness of consciousness
Contents of consciousness	Physical	Physical features
	Active	Action skills
	Psychological	Traits and values
	Social/relational/collective	Social role and membership, reputation, relationship to others

^a^Five levels^24^ in contrast to a level zero corresponding to a level of confusion with absence of self-consciousness

Self-consciousness can be impaired in one or several of its components (identity, body, etc.). Self-recognition, and notably self-image recognition, can be disturbed in various disorders, including neurodegenerative disorders (such as dementia) and neurodevelopmental disorders (such as schizophrenia and Autism Spectrum Disorder).^29^ Studying impairments in self-consciousness and self-recognition may open important perspectives, especially for early diagnosis of schizophrenia and the development of adapted therapeutic strategies. However, a limit of such phenomenological inquiry remains the detection of these disturbances that relies on patients’ verbal reports. These patients’ reports should indeed be interpreted with caution, especially since body self is related to non-verbal aspects of consciousness.^30^ Thus a challenge consists in finding a way to quantify objectively such self-disturbances in schizophrenia with a non-verbal approach.^31^ As underlined by Tordjman and Mailhes,^32^ self-image development might be a good indicator of the evolution of the self-consciousness process, especially through self-image recognition in the mirror. The mirror was already used to observe pathological self-perception in mental disorders. Salem Shentoub^33^ was the first to report disturbances of self-image recognition in the mirror in intellectually disabled children. François Achille Delmas^34^ and Paul Abely^35^ both described at the onset of schizophrenia the “mirror sign,” referring to the need of certain individuals with schizophrenia to observe themselves frequently and during a long time when facing a reflecting surface.

A new paradigm developed by Thirioux et al.,^36^ based on the alter ego system designed and programmed by Moritz Wehrmann,^36^ allows specifically self–other differentiation and self-consciousness to be explored through self–other image recognition in the mirror. This paradigm can be used to study self-image recognition impairment in schizophrenia.

The objective of the present study was to examine self–other recognition in EOS and AOS individuals compared to typically developing controls (TDC) based on a new experimental paradigm using a mirror system and to examine effects of intermodal sensory perception on self–other differentiation.

## Results

### Demographic and phenotypic characteristics

The data from one EOS male and his matched control were excluded due to the impossibility to complete the recognition task, given its duration (around 1 h). Out of the 11 remaining EOS individuals (6 individuals with VEOS and 5 individuals with EOS), 7 individuals were exclusively medicated with atypical antipsychotics (4 individuals with VEOS and 3 individuals with EOS), 1 individual with EOS was only treated with benzodiazepine, and 3 individuals (2 individuals with VEOS and 1 individual with EOS) were not treated with any psychotropic medications at recruitment and testing given their long-term stabilized clinical state and their young age (age 12, 16, and 21 years). The atypical antipsychotics taken by patients with schizophrenia were associated with first-generation antipsychotics (such as loxapine or cyamemazine) for three individuals with VEOS. All AOS patients were medicated with atypical antipsychotics. The cognitive assessment using the Raven’s Matrices was not possible for one AOS individual due to the patient’s opposition. No EOS and AOS individuals were intellectually disabled according to the World Health Organization definition criteria based on a Total IQ (intelligence quotient) <70 (probably due to the recruitment of outpatients with social insertion and all stabilized at testing). Demographic and clinical characteristics of the 11 remaining EOS patients and 15 AOS patients are presented in Table 2.

**Table 2:** Demographic and clinical characteristics of the patients with early onset schizophrenia (EOS) and adult onset schizophrenia (AOS) included in the study

	EOS patients (*N* = 11)		AOS patients (*N* = 15)	
	Mean	SD	Mean	SD
Age at testing	16.7	6.8	36.8	7.6
Age at schizophrenia onset	10.7	5.2	22.7	5.3
Behavioral assessments				
BPRS	58.5	8.3	49.6	13.0
PANSS	99.5	11.4	71.2	17.0
Chlorpromazine equivalents of antipsychotic medications (mg)	143.2	262.6	491.2	393.2
Cognitive functioning				
Wechsler				
Total IQ	106	25		
VCI	110	21		
PRI	100	23		
WMI	97	20		
PSI	95	26		
Raven class				
1				3/14
2				1/14
3				3/14
4				2/14
5				4/14
6				1/14

Note: Behavioral assessments: *BPRS* Brief Psychiatric Rating Scale, *PANSS* Positive and Negative Syndrome Scale; Wechsler: *IQ* Intelligence Quotient, *VCI* Verbal Comprehension Index, *PRI* Perceptual Reasoning Index, *WMI* Working Memory Index, *PSI* Processing Speed Index; Raven: *Class 1* IQ > 130, *Class 2* IQ confidence interval [120–130], *Class 3* [110–120], *Class 4* [100–110], *Class 5* [90–100], *Class 6* [80–90], *Class 7* IQ < 80

### Main outcome analysis

The comparison of the results for the recognition task in the different conditions between individuals with schizophrenia and TDC are presented in Tables 3a–3c.

**Table 3a:** Comparison of the results for the recognition task in the four conditions between individuals with early onset schizophrenia (*N* = 11) and typically developing controls (*N* = 11)

	Individuals with schizophrenia (*N* = 11)		Typically developing controls (*N* = 11)		*z* Absolute value	*p* Value
	Mean	SD	Mean	SD		
M1 *Visual—alone (reference condition)*	110.9	20.0	128.5	16.9	2.21	0.03
M2 *Visual—alone*	122.4	10.4	138.8	17.1	2.51	0.01
M1 *Visual—Movement (raising forearms)*	110.9	18.6	136.4	31.8	2.06	0.04
M2 *Visual—Movement*	117.3	14.9	132.7	19.0	2.08	0.04
M1 *Visual—Touching the abdomen*	113.6	30.2	120.9	20.6	0.66	0.51
M2 *Visual—Touching the abdomen*	115.5	15.7	123.6	26.6	0.90	0.37
M1 *Visual—Smiling*	115.5	23.4	126.4	20.7	1.04	0.30
M2 *Visual—Smiling*	116.4	11.9	127.3	17.5	1.44	0.15

**Table 3b:** Comparison of the results for the recognition task in the three conditions between individuals with adult onset schizophrenia (*N* = 15) and typically developing controls (*N* = 15)

	Individuals with schizophrenia (*N* = 15)		Typically developing controls (*N* = 15)		*z* Absolute value	*p* Value
	Mean	SD	Mean	SD		
M1 *Visual—alone (reference condition)*	106.4	21.9	133.1	18.2	3.07	0.002
M2 *Visual—alone*	122.9	18.4	140.2	9.6	3.13	0.002
M1 *Visual—Movement (raising forearms)*	107.3	28.1	126.7	23.2	1.90	0.057
M2 *Visual—Movement*	127.3	22.8	126.7	12.3	1.62	0.106
M1 *Visual—Touching the abdomen*	106.7	29.2	124.0	23.8	1.65	0.10
M2 *Visual—Touching the abdomen*	128	14.7	135.3	11.9	1.6	0.110

**Table 3c:** Comparison of the results for the recognition task in the three conditions between individuals with schizophrenia (*N* = 26) and typically developing controls (*N* = 26)

	Individuals with schizophrenia (*N* = 26)		Typically developing controls (*N* = 26)		*z* Absolute value	*p* Value
	Mean	SD	Mean	SD		
M1 *Visual—alone (reference condition)*	108.3	19.7	131.1	18.8	3.74	<0.001
M2 *Visual—alone*	122.69	17.5	139.6	9.8	4.0	<0.001
M1 *Visual—Movement (raising forearms)*	108.9	29.2	130.8	21.5	2.79	0.005
M2 *Visual—Movement*	123.1	21.5	135.0	13.3	2.54	0.011
M1 *Visual—Touching the abdomen*	109.6	25.7	122.7	26.2	1.69	0.091
M2 *Visual—Touching the abdomen*	122.7	21.1	130.4	14.6	1.40	0.161

**Fig. 1:**
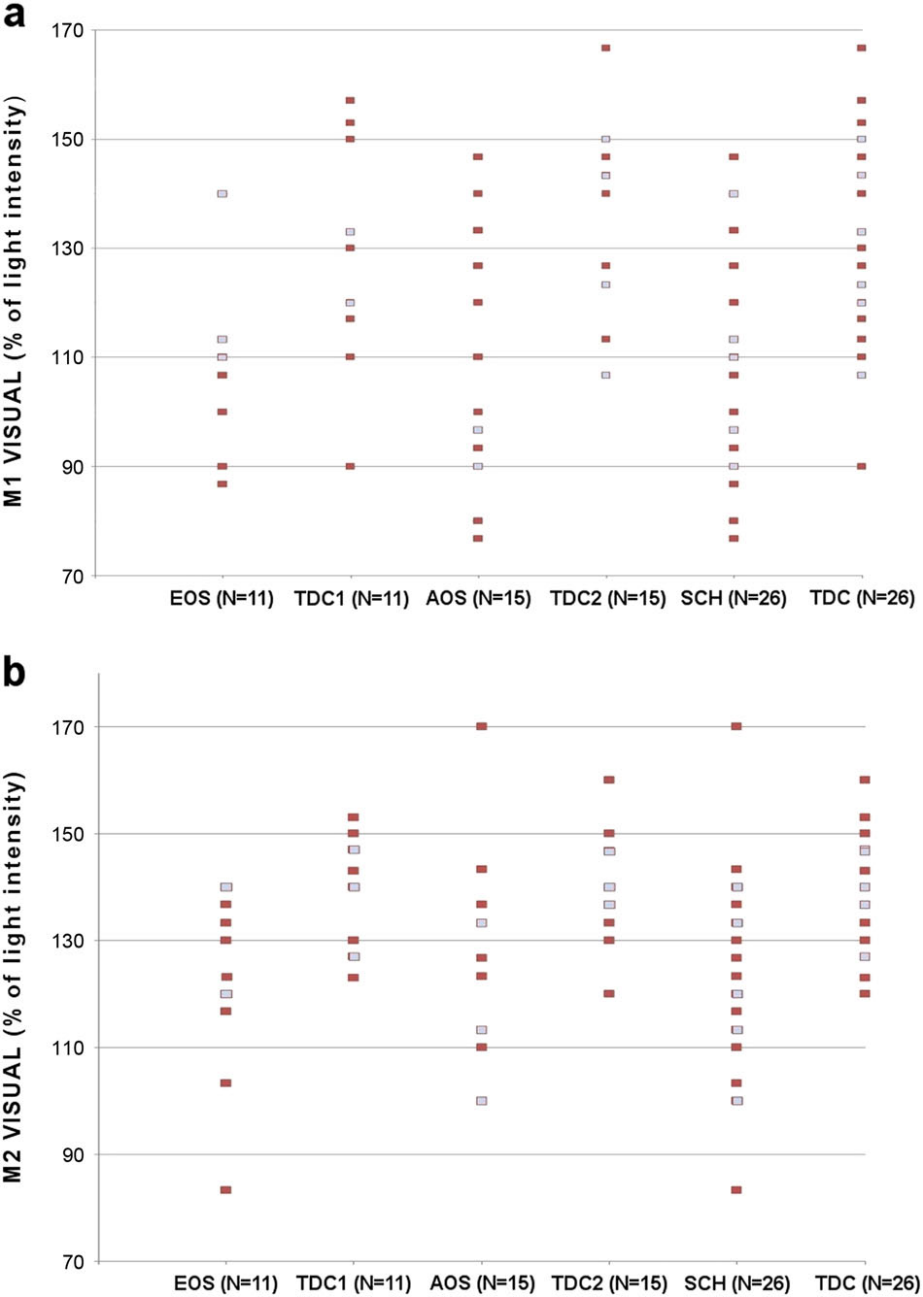
Distribution plots of M1 and M2 levels in the *visual—alone* reference condition for individuals with schizophrenia (EOS, AOS, and EOS+AOS) and typically developing controls. **a** M1 levels and **b** M2 levels are indicated by squares. Pale squares indicate more than one individual with the same value, whereas dark squares indicate only one individual with the value. EOS individuals with early onset schizophrenia, AOS individuals with adult onset schizophrenia, TDC1 typically developing controls matched with EOS individuals, TDC2 typically developing controls matched with AOS individuals, SCH total group of individuals with schizophrenia (EOS+AOS), TDC total group of typically developing controls (TDC1+TDC2)

In EOS and AOS individuals compared to controls (Tables 3a and 3b), M1 and M2 levels were significantly lower in the *Visual—alone* condition (this result was still significant after Bonferroni correction), indicating that patients with schizophrenia recognize their own image earlier than controls when it appears in the mirror in the *other-condition* and that they recognize the other person’s image later when it appears in the mirror in the *self-condition*. Also, in EOS individuals compared to controls (Table 3a), M1 and M2 levels were significantly lower in the *Visual—Movement* condition, but this result was not significant after Bonferroni correction. There was no significant difference between the EOS and control groups for the *Visual—Smiling* and *Visual—Touching the abdomen* conditions. The comparison of the EOS and AOS groups did not show any significant differences, therefore the results of the total group of AOS and EOS individuals are presented in Table 3c. These results were similar to the ones observed in the EOS and AOS groups compared to controls. Due to increased sample size, the statistical power was higher, and the significance of the *p* values was improved (Table 3c). Distribution plots of M1 and M2 levels in the *Visual—alone* reference condition for individuals with schizophrenia (EOS, AOS, and EOS+AOS) and TDC are presented in Fig. 1a, b.

### Effects of intermodal sensory stimulation on self–other recognition

In individuals with schizophrenia (EOS, AOS, and total EOS+AOS groups), there was no significant difference between the results for the recognition task in the reference condition (*Visual—alone)* and the results obtained in the other conditions, indicating that there was no significant effect of intermodal sensory stimulation on visual self-recognition. However, in the control group matched with the EOS group, the M2 level was significantly lower in certain conditions where intermodal sensory stimulation applied (*Visual—Touching the abdomen*: *z* = 2.30, *p* = 0.022; *Visual—Smiling*: *z* = 2.15, *p* = 0.032) compared to the reference condition (*Visual—alone)*. These results indicate that there is for TDC a delayed recognition of the other person's image when the visual recognition was associated with self-touching the abdomen or smiling. Inversely, the M1 level was significantly higher in the *Visual—Movement* condition than in the *Visual—alone* condition (*z* = 2.68, *p* = 0.007), indicating a delayed self-recognition in TDC matched with EOS patients when raising forearms was associated with visual perception compared to visual self-recognition alone. In the control group matched with the AOS group, the M1 level was significantly lower in the *Visual—Touching the abdomen* condition than in the *Visual—alone* reference condition (*z* = 2.106, *p* = 0.035). These results indicate that there is for the TDC an earlier recognition of self-image when the visual recognition was associated with self-touching the abdomen. Finally, in the total control group matched with the AOS and EOS groups, the M1 and M2 levels were significantly lower in the *Visual—Touching the abdomen* condition than in the *Visual—alone* reference condition (*z* = 2.403, *p* = 0.016; *z* = 2.776, *p* = 0.006, respectively).

### Effects of descriptive variables in individuals with schizophrenia

There was no significant correlation between the results of the recognition task and EOS or AOS patients’ age at testing, the Brief Psychiatric Rating Scale (BPRS) and Positive And Negative Syndrome Scale for schizophrenia (PANSS) scores, or the antipsychotic medication dose prescribed. Also, there was no significant relationships between the levels of cognitive functioning of EOS or AOS patients and the results of the recognition task in the *Visual–alone* and intermodal sensory conditions (*Visual—Touching the abdomen* and *Visual—Movement* conditions). Further analysis showed significant negative correlations in the EOS individuals between M2 levels in the *Visual—Smiling* condition and IQ scores (Total IQ: *r* = −0.78, *p* = 0.007; Verbal Comprehension Index: *r* = −0.78, *p* = 0.013; Perceptual Reasoning Index: *r* = −0.79, *p* = 0.004; PSI: *r* = −0.80, *p* = 0.003; Working Memory Index: *r* = −0.66, *p* = 0.028).

## Discussion

The main findings confirmed the hypothesis that individuals with schizophrenia (EOS and AOS), when compared to TDC, have difficulties to decenter from their own image with both an earlier self-recognition and a delayed other-recognition during the recognition task; also, compared to the *Visual–alone* condition, there was no significant effect of intermodal sensory stimulation on self–other recognition in individuals with schizophrenia (EOS, AOS, or EOS+AOS), whereas significant effects were observed in TDC.

The results are in line with observations and studies reported by several authors^37^ suggesting that self-disorder is one of the main dimensions of schizophrenia. The meta-analysis by Hur et al.^37^ suggested more particularly altered bodily self-consciousness and frequent agency impairments with increased rather than decreased self-consciousness. However, this self-focused functioning concerning self–other image recognition might be related to anxiety and defense mechanisms in individuals with schizophrenia^38^ to cope with decreased self-consciousness (in fact, the opposite of increased self-consciousness) with a weakening of the sense of self, especially the body self, leading patients to overinvest visual self-recognition. The existence of dysmorphophobia in individuals with schizophrenia and reports by some of them of daily use of mirrors at home to verify that “they are still here,” as they say, support this hypothesis.^32^ It is noteworthy that self-referential processing abnormalities in schizophrenia have been linked to cognitive control impairments^39^ and a dysfunction of large-scale brain networks such as the salience network and the resting mode default.^40,41^ Also, the present findings suggest self-focused functioning in schizophrenia with difficulties to take into consideration others, which might be relevant of a deficit in theory of mind. The results obtained in the present study could also be interpreted as a difficulty for the patients to inhibit their own perspective and change reference frames to adopt the reference of the other.^42,43^

The absence of effects of sensory intermodal stimulation on visual self–other recognition in schizophrenia might be related to the preponderant role of visual perception compared to other sensory perception. This hypothesis is supported by the Thakkar et al. study^44^ showing that visual stimulation increased self-perception more than tactile stimulation in individuals with schizophrenia compared to healthy controls. However, another hypothesis might be the existence of altered mechanisms of intermodal sensory integration in schizophrenia^45,46^ leading to similar results in the *Visual—alone* condition compared to the *Visual—Touching* and *Visual—Movement* conditions for individuals with schizophrenia, whereas perception of self is enhanced in TDC when cued by multiple sensory stimuli.^47^ The salience network plays a central role in cognitive control by integrating sensory input to guide attention.^48^ Its alteration reported in schizophrenia^48^ might also explain the absence of significant effects of intermodal sensory stimulation observed in this study on the results of the recogition task for individuals with schizophrenia.

In typically developing controls, visual–tactile stimulation (*Visual—Touching the abdomen*) was significantly associated with delayed other-recognition and earlier self-recognition compared to the *Visual—alone* condition, suggesting that visual–tactile stimulation helps the individual to be self-centered. Touch is usually described as an essential sensory modality, the first one to appear in fetal development (from 10 weeks of pregnancy for oral, peri-oral, and palmar tactile reactivity to 15–16 weeks of pregnancy for the rest of the body), whereas the visual sensory system is the last one to appear (from the third trimester of pregnancy to complete maturity only after birth).^49,50^ Tactile stimulation may have a specific important role in early development of self-consciousness by helping, through haptic memory, the individual during early development to build his/her identity—his/her self—especially body self.

Similarly, as observed for the *Visual—Touching the abdomen* condition, the *Visual—Smiling* condition was significantly associated with delayed visual other-recognition in the control group matched with EOS patients. However, smiling, particularly social smiling, involves other aspects than sensory modalities such as affective and cognitive functioning. It is noteworthy that, even in EOS individuals, significant correlations were observed between high levels of cognitive functioning (Total IQ as well as the four IQ subscales) and delayed other-recognition in the *Visual—Smiling* condition, suggesting that *Visual—Smiling* condition strengthens self-centered functioning through cognitive skills and mechanisms.

In contrast with the *Visual—Touching* and *Visual—Smiling* conditions, the *Visual—Movement* condition (visual perception combined with kinesthetic stimulation provoked by raising forearms) was significantly associated with delayed self-recognition compared to the *Visual–alone* condition in the control group matched with the EOS group, suggesting that movement might help typically developing individuals to decenter from themselves and to interact with others.

Also, the absence of significant effects of patients’ age, level of cognitive functioning, type of schizophrenia (similar results were observed in EOS and AOS individuals), and severity of schizophrenia symptoms (assessed on BPRS or PANSS) on the results of the recognition task in the *Visual—alone* and intermodal sensory conditions (*Visual—Touching the abdomen* and *Visual—Movement* conditions), suggest that self–other recognition disorders might be a stable trait characteristic of schizophrenia; this is consistent with large-scale brain network alterations in schizophrenia. Furthermore, self–other recognition disorders might be a common dimension shared by VEOS, EOS, and AOS given that all individuals with schizophrenia (VEOS, EOS, and AOS) showed significantly lower M1 and M2 levels than TDC in the *Visual—alone* reference condition. It suggests that VEOS, EOS, and AOS might be part of a same continuum rather than being considered as different types of schizophrenia.

Some limitations of the study should be acknowledged. The present study was conducted on small size samples, given the particularly low EOS prevalence. Future studies are requested to duplicate the findings on larger samples. In addition, there might be a bias of participants’ oral responses influencing each other. To decrease this possible bias for individuals with schizophrenia, they were asked to answer systematically first during the recognition task. The use of response button boxes has been initially discussed to control the bias of participants’ oral responses, but besides the difficulty to use manual buttons in individuals with schizophrenia, oral responses seemed to reassure these patients during the recognition task by maintaining relations with others and by comforting them in their identity and self-affirmation. Also, aleatory rather than linear and progressive variations of light intensity was initially discussed to control a possible bias of habituation in participants, but brutal changes of light intensity and therefore of images in the mirror could be stressful for patients with schizophrenia. Similarly, the order of presentation of the tasks was not randomized and this could lead to carry over effects. However, the order of the tasks was based on their level of complexity, which facilitated greatly the understanding of instructions in patients with schizophrenia. Furthermore, there was no significant time effect on the results of the recognition task in individuals with schizophrenia as well as TDC, which allows to reduce possible carry over and learning effects of the tasks. Finally, it was not possible to test thoroughly the *Visual—Smiling* condition in EOS individuals due to difficulties with smiling for some of them.

In conclusion, the present study suggests that self-image consciousness disturbances, and more specifically self–other recognition impairments, might be a possible endophenotypic trait of schizophrenia. The double mirror Alter Ego paradigm could be an interesting tool to study self–other recognition impairments in self-consciousness disorders in general and neurodevelopmental disorders such as schizophrenia or Autism Spectrum Disorder in particular. Indeed, several authors consider schizophrenia and autism as neurodevelopmental disorders, and more specifically as social developmental disorders closely linked to self-consciousness disorders.^51^ Self–other face identification in the mirror can be used to improve bodily self-consciousness and sustain self–other differentiation in these disorders. The double mirror system might be useful for early diagnosis, follow-up, and therapeutic perspectives based on cognitive remediation helping individuals with schizophrenia or other social developmental disorders to improve self–other differentiation.

## Methods

### Participants

The study was conducted on 12 individuals with EOS (mean age: 18.0 ± 7.7 years, 9 males and 3 females) matched on age and sex with 12 TDC (mean age: 17.7 ± 7.6 years, 9 males and 3 females). The two groups did not differ significantly with respect to age, age at testing, and sex. The EOS group was divided in two sub-groups depending on the age at onset of schizophrenia: VEOS (onset before age 13 years; *n* = 6) and EOS (onset after age 13 and before age 18 years; *n* = 6). The study was also conducted on 15 individuals with AOS (mean age: 36.8 ± 7.6 years, 13 males and 2 females) matched on age and sex with 15 TDC (mean age: 34.3 ± 9.0 years, 13 males and 2 females). The two groups did not differ significantly with respect to age, age at testing, and sex.

Individuals with schizophrenia (EOS and AOS) were recruited from French outpatient day-care facilities and were all stabilized at recruitment and testing. The diagnosis of schizophrenia was made according to the DSM-5 and ICD-10 criteria by two independent psychiatrists. Other psychiatric disorders were ruled out using the Mini-International Neuropsychiatric Interview (MINI). The TDC individuals matched with EOS patients were recruited from local schools and the TDC individuals matched with AOS patients were recruited from local universities (medical and nurse schools). TDC were determined to be free of any significant developmental, neurological, or psychiatric disorder based on the MINI and a medical examination. A family history of psychotic disorders in a first-degree relative was also ruled out and was an exclusion criterion in the TDC group. Finally, no participants had visual deficits requiring vision correction (eyeglasses or contact lens). The protocol was approved by the ethics committee of Bicêtre hospital, and written informed consent was obtained from the participants and their parents.

### Cognitive and behavioral assessments

Cognitive functioning of patients with EOS was assessed by a psychologist using the age-appropriate Wechsler intelligence scales (WISC-IV for 9 individuals aged <16 years, WAIS-IV for 3 individuals aged >16 years). Cognitive functioning of patients with AOS was assessed by a psychiatrist using the Raven’s Progressive Matrices, which is a short (20 min) nonverbal intelligence test. It was difficult to use the age-appropriate Wechsler intelligence scale (WAIS-IV) in the AOS group, given that this assessment has a duration of 2 h with a patient and most of the AOS individuals were reluctant for such a duration. In both EOS and AOS groups, symptoms were assessed with the BPRS and PANSS. The PANSS is a 30-item scale used to assess the severity of positive and negative symptoms and general psychopathology in individuals with schizophrenia. The BPRS is a clinician-rated measure that assesses psychiatric symptoms such as somatic concern, suicidality, unusual thought content, and suspiciousness. The 18-item BPRS was used in this study.

### The double mirror

**Fig. 2:**
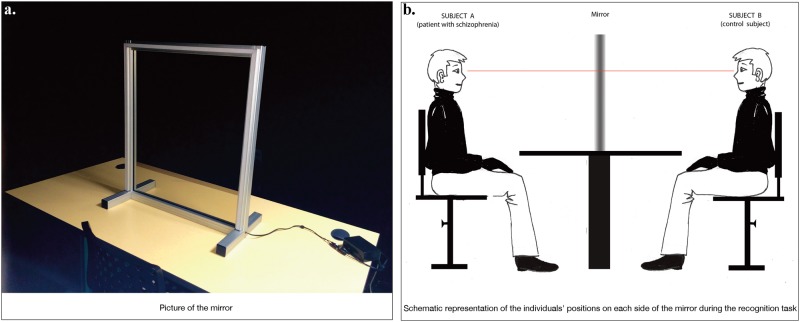
The experimental setting of the double mirror. **a** The experiment took place in an entirely darkened enclosed area that was set up inside the testing room in the Research Center of Hospital Guillaume Regnier in Rennes. The mirror was set up on top of a rectangular table. The patient with schizophrenia and his/her matched control were sitting facing each other on both side of the double mirror. **b** Both participants were wearing a black long-sleeved shirt, a black turtle neck, and black gloves to lessen any possible parasite visual stimulation. They were asked to look straight into each other's eyes and to focus only on the face of the other person. Adjustable chairs allowed to align the participants' eyes. No participants had eyeglasses that would have been objects interfering with the recognition task

In this experiment, we used a new double mirror paradigm based on the Alter Ego System, which was designed by Moritz Wehrmann and used for the first time by Alain Berthoz as an experimental tool for self–other interaction studies in healthy participants.^36^ It was later used in an experimental protocol by Foucaud Du Boisgueheneuc to study self-recognition disorders in Alzheimer patients (article in preparation). This double mirror paradigm is therefore used for the first time in patients with schizophrenia. The experimental setting is described in Fig. 2a, b. This consists of a semi-transparent double mirror (70 cm × 50 cm × 0.4 cm; height×width×depth) with a set of white computer-controlled light emitting diodes (LEDs) fixed on the frame of the mirror on both sides. These LED sets can emit continuous lighting at different intensities, either separately (i.e., LEDs turned on for only one side of the mirror) or simultaneously (i.e., LEDs turned on for both sides of the mirror). This system enables generating different self-face and other-face perceptual conditions when two A and B individuals are facing either side of the mirror. If the LEDs are turned on for subject A's side, whereas the LEDs are turned off for subject B's side, A can see his/her own face reflected in the mirror but without seeing B's face through the mirror. This perceptual condition is referred by Thirioux et al.^36^ as the *self-condition*. Using this same lighting mode, subject B can see subject A's face through the mirror (as through a transparent window) but without seeing his/her own reflection. This perceptual condition is referred by Thirioux et al.^36^ as the *other-condition*. When both sets of LEDs are on, the reflections of subject A's and subject B's faces are merging in the mirror, making it potentially difficult for the individual to recognize his/her own face. The higher the light intensity is, the more visible is the image of an individual in the mirror.

### Procedure

The experimental procedure had a duration of approximately 1 h and was divided into two parts: the visual task and the intermodal sensory stimulation task

#### The visual task

During the first part, which will be called the *Visual—alone* condition, inspired from the experimental protocol previously used by Foucaud Du Boisgueheneuc in Alzheimer patients, the light intensity of the LEDs sets was gradually increased or decreased on both sides of the mirror, one side at a time, so that both individuals found themselves alternately in the *other-condition* or the *self-condition*.

In the procedure, the patient starts in the *other-condition* and then switches to the *self-condition*, as follows:

→ In the *other-condition*, the patient starts seeing the control's face through the mirror but without seeing his/her own face; then the patient's own image appears more and more in the mirror following the light intensity;

→ In the *self-condition*, the patient starts seeing his/her own face reflected in the mirror but without seeing the control's face through the mirror; then the control's image appears more and more in the mirror following the light intensity.

The control subject experiences the same procedure except that he/she starts with the *self-condition* and switches after to the *other-condition*.

**Fig. 3:**
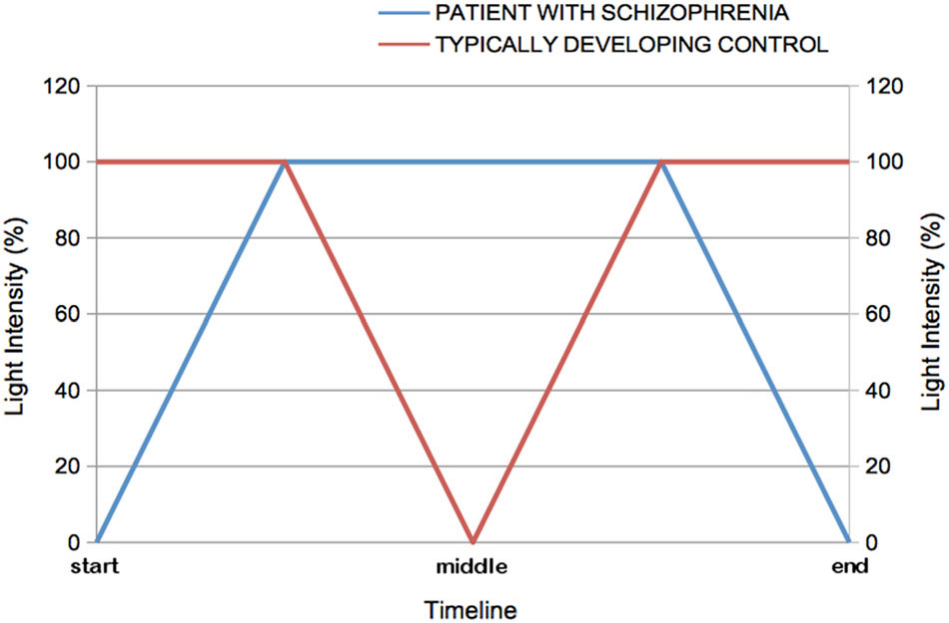
Simultaneous variations of light intensity on each side of the mirror over the time span of the procedure. At the beginning of the task, the light intensity is at 100% for the TDC individual (corresponding to a total/complete mirror effect), whereas the light intensity is at 0% for the patient (corresponding to a total/complete transparent window). Then the light intensity is progressively increased by 10% steps for the patient to reach 100% on both sides (the light intensity remains at 100% for the TDC individual). In other words, the patient's image appears progressively and is combined more and more with the TDC's image. At this point, the light intensity is progressively decreased by 10% steps for the TDC, whereas the light intensity remains at 100% for the patient (mirror effect). In other words, the TDC image fades away progressively up to its total disappearance when the light intensity drops down to 0%. The reverse procedure is then used to go back to the initial configuration (100% of light intensity corresponding to a total mirror effect for the TDC individual and 0% of light intensity corresponding to a completely transparent window for the patient). Therefore, each stimulus of identical light intensity is presented twice to the participants during one passage back and forth

The simultaneous variations of light intensity on each side of the mirror for the patient with schizophrenia and his/her matched control are presented in Fig. 3.

After every change of light intensity, both subjects were asked a simple question: “who do you recognize most in the mirror?” The expected response was either “me” or “[the other person's first name].” The question was always addressed to the individual with schizophrenia first. The *visual task* was repeated three times, and therefore each stimulus of identical light intensity was presented six times to the participants in this first task (three passages back and forth as described in Fig. 2). There was a systematic 10 min pause at the end of the first part. Drinks and snacks were offered during that break.

#### The intermodal sensory stimulation task

In this second part, the same procedure was applied but with the added instruction that after every change of light intensity and before answering the question “who do you recognize most in the mirror?,” both participants were asked to execute a simple task.

There were three different tasks, testing different *intermodal sensory stimulation* conditions. The participants were successively tested for each of these separate tasks (each stimulus of identical light intensity was presented twice to the participants during one passage back and forth as previously described in the *visual task*). The three tasks were administered always in the same order based on their level of complexity:

1. Raise forearms at face level (*Visual—Movement* condition);
2. Touch abdomen with the palms of both hands (*Visual—Touching the abdomen* condition);
3. Smile without showing teeth (*Visual—Smiling* condition).

These tasks allowed to study the effects of intermodal sensory perception (such as visual–kinesthetic perception or visual–tactile perception) on self-recognition. In the study on EOS patients, the visual task and all three intermodal sensory stimulation conditions were tested. In the study on AOS patients, the procedure was limited to the visual task and tasks 1 and 2 of the intermodal sensory stimulation conditions (*Visual—Movement* condition and *Visual—Touching the abdomen* condition), given the need to shorten in AOS individuals the duration of the procedure as previously mentioned for the cognitive assessment.

### Main outcome

The main outcome was the light intensity levels: the level M1 was the threshold corresponding to the ability of the individual to recognize his/herself when his/her own image appears progressively in the mirror in the *other-condition*; inversely, the level M2 was the threshold corresponding to the ability of the individual to recognize the other's image in the mirror when this image appears progressively in the mirror in the *self-condition*. To summarize, the lower the M1 or M2 level was, the more it reflected the individual's difficulty to decenter from his/her own image.

### Statistical analysis

After each step, the participant's responses were recorded. The analysis of the main outcome variable was conducted by comparing the M1/M2 level (expressed in percentage of light intensity) between individuals with schizophrenia and TDC. The Kolmogorov–Smirnov test indicated that this variable was not normally distributed; thus the non-parametric Mann–Whitney test was used to compare M1 and M2 levels between the two groups in each condition (*Visual—alone*, *Visual—Movement*, *Visual—Touching the abdomen*, and *Visual—Smiling*). The effects of intermodal sensory stimulation on the M1 and M2 levels observed during the recognition task were studied by using the non-parametric Wilcoxon test to compare for each group the results obtained in the reference condition (*Visual—alone*) and the results obtained in the intermodal sensory conditions (*Visual—Movement*, *Visual—Touching the abdomen*, and *Visual—Smiling*). Also, Spearman correlation analyses were performed to study relationships between results of the recognition task (M1 and M2 levels) and schizophrenia phenotypic characteristics (including not only age characteristics and schizophrenia severity but also medication status and level of cognitive functioning). Bonferroni correction was used to control for type I errors. Statistical analyses were performed using SAS, version 8.2.

## Data Availability

The data analyzed during the current study are not publicly available but are available from the corresponding authors on request.
